# “Cover up your arms, you’re triggering people”: A Mixed‐Methods Investigation of Shame in those who Self‐Injure

**DOI:** 10.1111/papt.12394

**Published:** 2022-04-11

**Authors:** Alexandra C. Brown, Cameron Latham, Adam N. Danquah, Brendan J. Dunlop, Peter J. Taylor

**Affiliations:** ^1^ Division of Psychology & Mental Health School of Health Sciences University of Manchester Manchester Academic Health Sciences Centre Manchester UK; ^2^ IMAGO Training Ltd. Wigan UK

**Keywords:** experience sampling methodology, mixed methods, non‐suicidal self‐injury, qualitative, self‐injury, shame

## Abstract

**Background:**

Shame can be a powerfully aversive emotion that is associated with a wide variety of mental health difficulties including non‐suicidal self‐injury (NSSI). This study used a novel mixed‐methods design (Qualitative Experiential Sequence Tracking; QUEST) to investigate the experiences of shame in a sample of individuals who self‐injure.

**Methods:**

Six participants received prompts to complete brief online diaries three times per day over a period of 2 weeks. These diaries captured information about the experience of negative emotions, especially shame. Participants then underwent an individualised qualitative interview about their experiences over the previous 2 weeks.

**Results:**

Thematic analysis suggested that participants experienced shame as a social and relational emotion. Further themes included shame being associated with feelings of failure, being trapped, dangerous or contaminated, and hidden or exposed. The phenomenology of shame, and coping with shame, were also themes. NSSI could occur as a response to shame, but often shame was triggered or exacerbated by the responses of others to NSSI.

**Conclusions:**

Consistent with previous research, shame was described as an aversive emotion occurring within interpersonal and broader societal contexts and involving a negative self‐focus. A lack of compassion or understanding in response to NSSI, or anticipation of negative responses from others often triggered more intense shame than the NSSI itself. Future studies could use QUEST methodology with more diverse samples or different populations to further investigate experiences of shame.

**Practitioner points**

- QUEST is a qualitative methodology combining diary and interview data
- Shame was commonly reported by people who self‐injure
- Participants felt trapped within shame by internal and external factors
- While shame sometimes triggered self‐injury, more often it was a consequence

## INTRODUCTION

Non‐suicidal self‐injury (NSSI), defined as intentionally and directly injuring one's own body tissue without suicidal intent (e.g., cutting, burning, scratching, biting; Klonsky, 2007; Muehlenkamp, 2005), is a prevalent health concern associated with distress and later risk of suicide (Arbuthnott & Lewis, 2015; Klonsky, 2007; Ribeiro et al., 2016; Richmond et al., 2015; Swannell et al., 2014). NSSI can be understood as a subtype of *self*‐*harm*, a term which covers both suicidal and non‐suicidal behaviours (National Collaborating Centre for Mental Health, 2004). Research suggests that there are a number of reasons why an individual may self‐injure, such as self‐punishment or to prevent suicide (for further detail see Klonsky, 2007); however, the most commonly endorsed function of NSSI is emotion regulation, particularly to reduce negative affect and arousal (Taylor et al., 2018). While emotion appears important, it is less clear if specific emotional states are more important than others in triggering or maintaining problems with NSSI.

One emotion that is associated with NSSI is shame (see review by Sheehy et al., 2019). Shame is an aversive, self‐conscious emotion, associated with a sense of the self as being flawed, inferior or defective in some way (Chou et al., 2018; Sheehy et al., 2019). It has been suggested that self‐injury may be motivated by desire to regulate or reduce feelings of shame (e.g., where self‐injury functions as a form of self‐punishment; Schoenleber et al., 2014; Sheehy et al., 2019). Relatedly, it has been proposed that NSSI may emerge as part of shame‐related coping scripts (in particular, those involving internalisation and magnification of such feelings), which may become reinforced through the repeated use of NSSI (Mahtani et al., 2018, 2019). Strong feelings of shame may also erode an existing drive to protect oneself from harm and so remove a key barrier to engaging in self‐injury (Hooley & Franklin, 2018). In a recent review and meta‐analysis, Sheehy et al. (2019) found that elevated levels of shame have been reported by those with a history of NSSI (*d* = 0.47) and shame is positively correlated with the frequency of NSSI (*r* = .24). The authors noted that the limited number of longitudinal studies makes it difficult to draw conclusions about the temporal characteristics of this relationship. The significant stigma that surrounds NSSI may mean that shame is also a consequence of the behaviour, in addition to being a cause (Staniland et al., 2021).

Studies using experience sampling methodology (ESM) further support the suggestion that feelings of shame may actively trigger experiences of self‐injury, by highlighting how such feelings peak and decline following NSSI (e.g., Armey et al., 2011). However, such quantitative approaches are still limited in their ability to provide a more in‐depth and individually nuanced understanding of how shame is experienced and processed by those who self‐injure.

Qualitative research provides another line of inquiry that may be well suited to explore the lived experience of shame among people who self‐injure and capturing their perceptions of whether this emotion is a cause or a consequence of their self‐injury. Qualitative research may therefore provide a better understanding of the individual experiences and processing of shame (e.g., Chapple et al., 2004; Rørtveit et al., 2010). While research (e.g., Harris, 2000; Stänicke & Haavind, 2018) suggests the potential importance of shame for people who self‐injure, we are not aware of studies that have focused specifically on the experience of this emotion within the context of self‐injury.

Traditional qualitative interviews alone may be limited where a person is being asked about specific emotional experiences that have occurred in the past and may not effectively capture psychological processes that occur “in‐the‐moment.” To overcome this challenge, we developed Qualitative Experiential Sequence Tracking (QUEST). This is a novel mixed‐methods approach that combines the use of multiple daily assessments to capture “in‐the‐moment” experiences (as in ESM; Palmier‐Claus et al., 2019) with tailored qualitative interviews. This then allows a deeper exploration of experiences picked up during the daily assessments. Other studies have used similar approaches, combining diary or ESM methods with interviews (Mort et al., 2005; Spillane & Hunt, 2010), although we are not aware of any past studies using such an approach to track and investigate a particular psychological phenomenon as in QUEST. A strength of ESM is that it captures experiences “in‐the‐moment,” limiting the impact of retrospective recall bias and affording responses greater ecological validity (Palmier‐Claus et al., 2019). QUEST drew on these strengths but placed them in a qualitative context. The ESM component of this design helped to capture specific experience within the moment, and then interviews further dissected these experiences. The diary data provided a focal point for the interview, and a way of supporting participants in discussing shame, which can be a challenging topic to discuss.

This study aimed to investigate the psychological processes that are associated with shame in those who engage in NSSI. Specifically, the study focused on how these feelings were triggered, how they were processed (i.e., made sense of and experienced) by the individual and how the person responded or attempted to cope with these feelings. The study aimed to elaborate on participants’ experiences across these three aspects of shame to better understand the psychological processing of this emotion and its ties to self‐injury.

## METHOD

### Design

The current study employed a novel, mixed‐methods design (QUEST) that combined qualitative interviewing with ESM. The protocol was pre‐registered on the Open Science Framework (osf.io/sd5gk).

### Participants

Participants were recruited from NHS Mental Health Services in the north‐west of England via referrals from mental health clinicians. Participants also self‐referred into the study through advertisements. Inclusion criteria were as follows: aged 16–25 years; owned a smartphone; had no difficulties understanding the English language and had engaged in NSSI on 5 or more days in the past year. Individuals would not be eligible if NSSI occurred exclusively during psychotic episodes, substance intoxication or be part of a pattern or repetitive stereotypies associated with a neurodevelopmental condition (e.g., Lesch‐Nyhan syndrome), as determined by self‐report during the screening interview. For the purposes of this study, self‐poisoning and eating disorder behaviours such as purging were not considered forms of NSSI, reflecting the DSM‐V criteria for NSSI disorder (APA, 2013). In line with Nelson (2017), recruitment of participants stopped when a sufficient depth of understanding had been achieved to allow for the team to theorise, which occurred after interviews with six participants. Participants were reimbursed with a £30 shopping voucher for their time.

### Measures

#### Baseline measures

The Experience of Shame Scale (ESS; Andrews et al., 2002) was used to assess specific areas of shame related to self and performance. This scale has good construct and discriminant validity, high internal consistency (Cronbach's *α* = .92) and a test–retest reliability score of *r* = .83 (Andrews et al., 2002).

The Test of Self‐Conscious Affect Version 3 short form (TOSCA‐3S; Tangney et al., 2000) was used to assess general levels of shame and guilt‐proneness, and externalisation. The current study used the norms given for female participants as the majority of participants identified as female (see Table 2). Participants answered 11 items each with three subquestions that address shame self‐talk, guilt self‐talk and “blaming others. This measure has demonstrated adequate reliability and acceptable to good internal consistency, although its internal consistency may be lower than expected as the tool uses a scenario‐based design (for an overview, see Broerman, 2018). The ESS subscales correlate highly with the TOSCA shame subscale (*r* = .51 to .61; Andrews et al., 2002).

The Patient Health Questionnaire‐9 (PHQ‐9; Kroenke et al., 2001) was used to assess low mood over the past 2 weeks. This measure has excellent internal reliability (Cronbach's *α* = .86 to .89) and good criterion and construct validity (Kroenke et al., 2001).

An adapted version of The Self‐Injurious Thoughts and Behaviours Inventory short form (SITBI; Nock et al., 2007) was used to gather information regarding history, frequency, methods and severity of NSSI. Questions surrounding frequency of NSSI (e.g., “How many times in the past year have you purposefully hurt yourself without wanting to die?”) were adapted to give a Likert‐type response format to avoid extreme guesses. This measure has good construct and concurrent validity, and strong inter‐relater and test–retest reliability (Nock et al., 2007).

#### ESM diaries

The ESM questions focused on recent experiences of shame. Question 1 asked participants: “Since the last text, have you felt bad about yourself or something you did?” Participants who responded positively to this were then asked to answer further questions around physiological symptoms, coping strategies and potential triggers and processes. A combination of closed and free response formats was used (see File S1 for copy of diary). The initial question was left deliberately broad, rather than asking specifically about shame, so as to avoid excluding experiences of shame that the participant may not have labelled as such. Participants were informed that if they answered “no” to question 1 that they did not have to complete the remainder of the questions for that assessment.

#### Qualitative interview

A generic interview template (see File S2) was tailored for each participant based on their ESM entries using a method of diary elicitation (Carter & Mankoff, 2005), whereby data from the electronic diaries were used to guide the interview. The researcher would identify two ESM entries as the focal points for each participant's interview. Copies of these entries were shared during the interview to aid this process. The entries selected were those that appeared consistent with feelings of shame (e.g., negative feelings about the self as flawed or inadequate), and that were rated as the most intense across the 2‐week ESM period. Where no shame was apparent in the ESM entries, the two entries with the other strongest emotion were selected. The semi‐structured interview was therefore used to further elaborate on and explore the instances described in the ESM data. Interview questions were then adapted to ask about these instances, for example, using the same language or terminology as the participant used in their ESM entry. During the interview, questions were asked about “self‐harm” rather than NSSI specifically, in order not to narrow responses and exclude potentially relevant phenomena.

#### Procedure

The project received NHS Research Ethics Committee approval. Individuals were either referred by clinicians or self‐referred (by contacting the research team), and then were invited to a telephone screening to determine eligibility. Eligible individuals were then invited to an initial meeting where informed consent was taken. During this meeting they completed the baseline assessment measures (ESS, PHQ9, SITBI and TOSCA‐3S) and received instructions on completion of the ESM diaries (based on Palmier‐Claus et al.’s best practice guidelines, 2011). They then underwent a “practice session” for the ESM diaries.

Participants then received prompts at three pseudorandom times per day for 14 days, via text messages providing them with a link to log on to the online survey platform using their smartphone. Participants had 1 h to start the assessment before the link was deactivated. The researcher telephoned the participants on day 2 of the ESM diaries to troubleshoot any technical issues and to discuss any potential negative or positive impact that completing the diaries was having on participants. Following completion of the diaries, the participants completed the qualitative interview, which was audio recorded. Five of six qualitative interviews were conducted face to face, however, one interview was conducted via telephone due to the emergence of the COVID‐19 pandemic. Interview length ranged from 25 to 54 min.

#### Data analytic strategy

Thematic analysis was used to analyse qualitative data from the interviews and diary entries. Thematic analysis was conducted in line with Braun and Clark’s (2006) six‐phased process: (1) identify items of potential interest; (2) generate initial codes; (3) search for themes; (4) review potential themes; (5) define and name themes and (6) produce a report. An inductive method was used to code the data and to generate themes. A critical realist epistemological stance was taken during analysis and interpretation as this was most consistent with the goals of the study. This stance assumes that psychological phenomena do have some external basis in reality outside of any single individual's interpretation, but that these phenomena are fuzzy and bounded by culture and context that also requires consideration (Kempster & Parry, 2011).

It is important to take culture and context into account for the participants and their data, but it is also important to consider these for the researcher analysing these data. The researcher who primarily analysed the data was a 29‐year‐old Caucasian, Atheist, heterosexual cisgender female. The researcher was originally from and currently based in the north‐west of England. They were educated to a Doctoral level in Clinical Psychology and had worked primarily in NHS mental health services since 2015. The researcher reflected that they possessed different social privileges, which likely influenced their experiences and expectations of the world, and their interpretations of it. They also reflected that their training as a Clinical Psychologist within the NHS could also influence how they interpret discussions and data regarding shame and NSSI. This includes the view of NSSI as a difficulty emerging from no one singular cause, but an interaction of social and psychological factors.

## RESULTS

### Participant characteristics

Table 1 gives the pseudonyms allocated to participants and their demographic characteristics. Participants were six individuals aged 17–23 years, with a mean age of 20.67 (*SD* = 3.01). Five participants identified as female and one participant identified as gender non‐binary. Fifty per cent (*n* = 3) of participants identified as White British, 33.3% (*n* = 2) identified as Asian British (Pakistani) and 16.7% (*n* = 1) identified as any other Mixed background. 66.7% (*n* = 4) of participants identified as heterosexual, 16.7% (*n* = 1) identified as bisexual and 16.7% (*n *= 1) identified as asexual. A further individual completed the initial meeting, however, was unable to continue with the study due to the COVID‐19 pandemic.

**TABLE 1:** Participant characteristics

Pseudonym	Age	Gender	Ethnicity	Sexuality	Co‐morbid diagnoses or difficulties aside from NSSI, depression and anxiety	Current mental health input
Katie	24	Female	White British	Heterosexual	Anorexia Nervosa, Body Dysmorphic Disorder, B&P, previous diagnosis of BPD and social phobia, query ASC but no diagnosis	Specialised Eating Disorder team
Charlotte	23	Female	White British	Heterosexual	ASC, psychosis^a^, reported B&P but no diagnosis	Community mental health team
Emily	23	Non‐binary	White British	Asexual	Current diagnosis of BPD	Community mental health team and supported living
Lucy	18	Female	Other Mixed background	Heterosexual	Reported restricting eating, B&P but no diagnosis	Psychiatry only
Zara	17	Female	Asian British (Pakistani)	Heterosexual	No diagnoses	Psychiatry only
Yasmin	19	Female	Asian British (Pakistani)	Heterosexual	No diagnoses	No specialist input

Note

All participants reported episodes of low mood and anxiety.

Abbreviations: ASC, autism spectrum condition; BPD, borderline personality disorder; B&P, bingeing and purging.

^a^ One participant had experiences of psychosis, primarily auditory hallucinations, but stated that they engaged in NSSI outside of these experiences and hence did not meet the study exclusion criteria. They therefore remained in the study.

All participants indicated that they had previously engaged in, or were currently engaging in, cutting or carving their skin. In the past year, three participants engaged in NSSI between 11 and 30 times, one between 31 and 50 times, one between 6 and 10 times and one between 0 and 5 times. Table 2 shows the participant scores for the ESS, TOSCA‐3S and PHQ‐9. The results indicate that, overall, the participants scored within the “often” range for shame self‐talk (TOSCA‐3S), and the moderately severe range for depression (PHQ‐9).

**TABLE 2:** Scores for the baseline measures

Participant	ESS	TOSCA−3S – shame self‐talk (category)	TOSCA−3S guilt self‐talk (category)	TOSCA−3S blaming others (category)	PHQ−9 (severity of reported depression)
Katie	64	35 (average)	31 (seldom)	26 (average)	18 (moderately severe)
Charlotte	45	34 (average)	40 (seldom)	16 (seldom)	13 (moderate)
Emily	86	38 (often)	47 (average)	21 (average)	7 (mild)
Lucy	91	44 (often)	46 (average)	21 (average)	23 (severe)
Zara	75	44 (often)	41 (seldom)	11 (seldom)	22 (severe)
Yasmin	51	40 (often)	47 (average)	16 (seldom)	10 (moderate)
Mean (*SD*)	68.7 (18.6)	39.2 (4.3; often)	42 (6.2; seldom)	18.5 (5.2; seldom)	15.5 (6.5; moderately severe)

Note

Ranges for TOSCA‐3S categories are as follows – shame (seldom = 0–26, average = 27–35, often = 36–55); guilt (seldom = 0–42, average = 43–48, often = 49–55) and “blaming others” (seldom = 0–20, average = 21–28, often = 29–55). Ranges for PHQ‐9 are as follows: no depression = 0–4, mild = 5–9, moderate = 10–14, moderately severe = 15–19 and severe = 20 or above.

### Experience sampling descriptive statistics

The total number of ESM entries completed was 152, meaning that approximately 60% of the possible maximum number of total entries were completed (maximum possible ESM entries per participant = 42). The mean number of total entries completed per participant was 25.33 (*SD* = 9.95). One participant reported engaging in NSSI during the study period, and five reported NSSI thoughts and/or urges. There were 70 entries where participants responded “yes” to question 1 (“since the last beep, have you felt bad about yourself or something you did?”; M per participant = 11.67, *SD* = 5.79), accounting for approximately 46.10% of the total entries completed. All participants completed the qualitative interview. In the diaries, three of the six participants described instances that they clearly labelled as shame (e.g., Katie described multiple instances of “shame” and feeling “ashamed”). In addition, there were other instances of feeling bad or disappointed in oneself (e.g., “feeling quite down about myself,” Emily) that might be suggestive of shame, although this was less clear. In some cases, the thoughts relating to a feeling suggested some degree of shame. For example, Lucy described feeling “annoyed” in relation to eating behaviour but then when asked about thoughts noted “ashamed, greedy, guilty.” Various other emotions were captured in the diaries including, most commonly, guilt, feeling down, feeling anxious, the urge to cut or feeling annoyed.

### Thematic analysis

Six themes were constructed from the data (interview and ESM diaries): (1) being trapped in shame, (2) failure, (3) hidden vs. exposed, (4) phenomenology of shame, (5) feeling dangerous to others and (6) controlling and combatting shame. Within the interviews, when referring to “self‐harm,” the behaviours participants described still typically met criteria for NSSI (e.g., self‐cutting without suicidal intent). This also reflects the colloquial use of the term “self‐harm” in the United Kingdom. Some participants did also describe other behaviours such as eating disorder that they saw as a form of self‐harm, and there were occasional explicit references to suicidal behaviour.

#### Being trapped in shame

Participants described being stuck in cycles of shame, or feeling “trapped” in shame, either by internal factors (e.g., through rumination, comparing themselves to others or maintained behaviours) or by others’ expectations of and responses (actual, perceived or anticipated) to them. Katie described shame as feeling different from other emotions in this regard: “If you feel ashamed, what can you do?.. it's not really [an emotion] that people teach you how to deal with, it's like, you can go online and say, like, how do you deal with anxiety, there are a hundred videos that will come up, erm, you try and say I feel ashamed of myself, what will come up is, you should love yourself, it's like, that's not very helpful.”

Emily described feeling trapped in a cycle whereby she tried to decrease shame by accepting her scars and wearing short sleeves, only to then be denigrated by others for this (“[she said], everyone's embarrassed by you… you need to cover up [the scars on] your arms.”), thereby further increasing shame and reverting to wearing long sleeves. Participants often reflected that others’ actual or anticipated responses to self‐injury triggered stronger shame than the behaviour alone, regardless of whether the behaviour was something they wanted to stop doing, and often kept them trapped in shame. Some participants would try speaking to others about their shame to relieve it but were often met with a lack of understanding or compassion, thereby exacerbating or re‐triggering shame.

Rumination related to shame could reinforce the feeling of being trapped or “stuck.” Emily said: “I just kept ruminating on it… it just kept going and going.” Lucy and Zara described thoughts and feelings “piling up,” and this exacerbating the intensity of emotion. Other people continued to feature in the rumination participants engaged in relation to shame, for example, rumination encompassed statements made by others, or the participants’ perceptions or anticipations of what others would think of them or their behaviour.

Breaking free of feelings of shame was difficult for participants. Lucy said: “I just can't bring myself to stop getting stuck in this cycle.” Attempts at reparative action through apology to others were described by some participants, and although this could relieve some guilt, it was often ineffective at reducing shame. Katie reflected on feeling stuck in shame with regards to self‐relational behaviour: “normally, if you've done something wrong, you feel ashamed, you say, ‘I’m really sorry’, and the person goes ‘I forgive you’ … but if it's something you're doing to yourself… there's nowhere for it to go.”

#### Feelings of failure

A sense of failure was reported across participants. This could be present across multiple domains, including perceived social, personal, academic or religious failures, (Yasmin: “in our religion we're not meant to be like in a relationship before marriage and stuff… so I’ve always, erm, known [my relationship] was wrong … I probably deserve [to be struggling, as a punishment]”), or feeling as though they were unable to achieve a goal (Katie: “I feel like I had every advantage and yet I still fucked it up”). For Emily, feelings of failure were closely associated with instances of self‐injury but were compounded by the way others responded: “I’ve just self‐harmed and I feel, like I’ve failed myself, but then when everyone else is looking at me and saying, well, well pretty much saying with the looks on their face, you've failed me, it makes it ten, times more worse.”

Participants often reported feeling as though they were disappointed in themselves, or had disappointed others, with a few participants lamenting “I’ve really messed up” or similar statements. Explicit feelings and thoughts of failure were present as part of processing, especially with regards to rumination, although these could also act as triggers for shame and could be experienced around coping strategies. For example, Charlotte felt it was her fault that safe self‐harm methods did not work because she had not practiced them enough. This sense of failure as part of shame often occurred when comparing self to others or thinking about social rank or hierarchy. For example, Katie said: “I don't wanna be at the bottom of the pile, and if I have to be at the bottom of the pile, I don't wanna be alive.” Thoughts about failure and being shameful to others were closely entangled for some. For example, in Emily's diary entry “I'm a failure and everyone hates me because of how I look. None of my friends invite me out anymore because I started wearing short sleeves and they are ashamed of me.”

#### Hidden vs. exposed

For most participants, there was a conflict or tension between wanting to be acknowledged and accepted, and also feeling the need to hide a lot of themselves for this to happen. Participants could feel invisible or dismissed or, conversely, overexposed and vulnerable. Emily noted in her diary “Had a few funny looks off people because I have my arms on show. They try not to make it obvious but you can just tell when they look.” In contrast, Katie described in her diary an instance of a doctor being very dismissive of her, triggering shame and an urge to harm herself.

Several participants described feeling they had to hide or change the aspects of their personality or behaviour that they felt ashamed of to be accepted and not feel alone. Zara said: “[my friends] know I’m quiet, but they don't know how I feel ‘cos I haven't told them… [I worry about] how they would see me.” Katie stated: “I want to be accepted, and I think, to be accepted I have to kind of fit into what other people deem to be acceptable… I just feel like if [others] knew what I was like they would hate me.”

Feeling ashamed was often an isolating experience, and was linked to feelings of isolation, loneliness, rejection and worthlessness. Emily said: “I got in bed, and no one came and checked on me all day, after I’d just sat there and had a full meltdown, so I felt like everyone was just blanking me, because I’d messed up, so it just added to the shame really… in the back of my mind, I was hoping someone would come up to my flat and check on me, and sort of save me, and no one did. So yeah [laughs].”

Participants often reported hiding their NSSI scars with long sleeves, as these scars could serve as “visible” sources of shame. Lucy engaged in both self‐injury and disordered eating but felt more shame around food and body image than scars (“I can cover up, er, scars, but I feel like I can't really cover up my weight that well”). Hiding behaviours could be due to shame around the act itself, for example, Charlotte stated that “[self‐injury is] a behaviour that I don't like.” However, for several participants it was others’ actual or anticipated responses to self‐injury that led to hiding behaviours/scars and shame. Katie said: “[I would only be ashamed of self‐injury] if someone made me feel ashamed. I don't say they made me feel ashamed as in it's their fault, but if their reaction kind of induced shame in me, whether that was their intention or not…” Non‐judgemental responses from others, and acceptance of self‐injury, could sometimes prevent shame, with Yasmin reporting positively that her family were “used” to her self‐injuring, that they trusted her to know how to go about it safely and that they would not try to prevent her from engaging in this unless they thought it was life threatening (e.g., if she became severely depressed).

In contrast, participants also reported feeling too visible or exposed, and some reported being looked at or stared at by others and feeling uncomfortable. Katie described an incident where she was fined for not paying the correct fare on public transport, and said “everyone was staring at me… I felt really, really, really ashamed.” Emily gave a statement that combined feeling exposed but also not acknowledged: “they were all just full on staring at me… every time I tried looking at them, they looked away.”

#### Phenomenology of shame

The phenomenology of shame was experienced and reported differently by participants. Shame was generally reported as an intense emotion, however, intensity could vary depending on the trigger and rumination (for some). Most participants reported that shame occurred alongside other emotions, including hopelessness, feeling defeated, futility, rejection, guilt, loneliness and anger. Shame was characterised by the desire to withdraw, hide away or “curl up in a ball.” It was also an emotion that was difficult to express or explicitly label (“harder to define, harder to articulate, harder to share with other people and harder to get rid of”; Katie).

The amount of time taken for shame to build varied between participants. Some described shame developing over a number of hours and others described “instant shame” occurring following a trigger. Identification of shame also varied across participants, Katie stated: “I don't even really notice it… if someone's asking me directly, I’m like … if I think about it objectively then yes I would think it was probably shame, but I don't really think of it in terms of, I feel ashamed… [if I think] ‘I hate myself’, I know that's connected to shame, so I’m like, ok, it's shame rather than sadness.”

For some shame might last for days, irrespective of how they may try to cope with this feeling, whereas others reported that shame occurs very intensely for a short period and then dissipates over time. However, many reported that residual shame could be present, and that shame could be easily re‐triggered, which could be experienced as shame lasting for a greater duration.

#### Feeling dangerous or harmful to others

Participants often reported feeling ashamed if they thought they might cause distress to others, with some feeling that this made them dangerous or harmful in some way. There was also a sense of feeling “contaminated.” Emily reported being told when wearing short sleeves: “put a jacket on, you're triggering people.” Charlotte reported that she felt she needed to wear long sleeves to hide her NSSI scars when working with children (“it's not fair on them, erm, and they don't know what they're looking at, and it's not fair to introduce them to it, ‘cos they're suggestible… I’ve got to be really careful… I can't put myself in a position where I could be that influence on a child”). Some participants reported feeling anger as a trigger for shame, reflecting that they felt they could not ever show anger to others (even if someone had done something to them that justified anger), as this was not socially acceptable and potentially destructive, and anger was often turned inward. Participants reported feeling responsible for others’ distress or negative emotions, for example, Charlotte said she sometimes felt ashamed about the damage she was doing to herself with NSSI but that this shame was less than the shame caused by worrying others. However, both Lucy and Emily also reflected that feeling shame about causing their loved ones distress was a protective factor against suicide.

#### Controlling and combatting shame

Two participants alluded to a battle or struggle around resisting self‐injury and feelings of shame. Emily described a “battle” against the shame she experienced from others when her self‐injury scars were visible, but also a battle against urges to self‐injure. Charlotte described a similar battle at times taking place with the urge to self‐injure. Katie commented on what could help her resist the urge to self‐injure when feeling shame: “whether I was able to distract myself, whether I’m able to sleep, whether I’m able to experience the emotion in a different way.” She also described feeling more in control over her shame when sharing it with someone she would not see again (i.e., the researcher), as opposed to sharing with someone she would see more regularly, as she would care what they thought of her and she would worry that what she had said would be passed on.

Although NSSI was sometimes used as a coping strategy to control or combat shame, more often than not shame was a perceived consequence of NSSI, and NSSI could exacerbate/re‐trigger the existing shame. The shame surrounding acts of self‐injury also extended to the experience of urges to self‐injury. Zara said, “I cut again I feel like a psycho, but if I don't cut, I still feel like a psycho, ‘cos I want to cut.”

Whether participants did or did not intend to cease their NSSI, they agreed that others’ actual or anticipated responses to self‐injury were the main triggers for shame. Four participants were contemplating or actively trying not to engage in NSSI, usually cutting, saying that this behaviour no longer gave relief for negative emotions (including shame) and could re‐trigger or exacerbate shame. Lucy said: “I just don't think [NSSI], it bothers me anymore… I feel like I want, like a rush to sort of snap me out of it, erm, like a rollercoaster, you go on it and like… you get it out and then … you get off, but, I’ve been on the rollercoaster that many times, it doesn't bother me anymore.” One participant (Zara) was in two minds about ceasing NSSI, she often used cutting as a coping strategy for shame and this gave some relief, but was concerned about the number of scars she had. Yasmin had no current plans to cease NSSI, as it did not trigger shame for her and her family were accepting of this coping strategy. She reflected that she may have felt differently about NSSI if others had responded negatively to this. Notably, Yasmin had the second lowest scores on the ESS, potentially reflecting the greater level of acceptance she experiences, although her shame self‐talk on the TOSCA was still “often.”

### General observations

Across the sample, participants scored as “average” or “often” for shame self‐talk on the TOSCA compared to lower levels of guilt self‐talk (see Table 2). This is mirrored in the qualitative data where experiences of shame (more so than guilt) were prominent in the responses. During thematic analysis of ESM and interview data, it became evident that shame almost exclusively occurred within a relational context, whether this was at a wider, societal level or through everyday interactions. Social context and interpersonal interactions were relevant in terms of triggers for, processing of and coping with shame. Charlotte said, “shame is a very social emotion… other [emotions] may be more isolated to me, whereas [shame is] very much in the context of everyone else.” Feelings of shame were also seen as being dictated by societal values, “[society says]…you should be ashamed if you've done something wrong” (Katie). Shame as a relational emotion cut across all other themes and therefore this is discussed within the themes presented below.

Despite others’ responses having an important impact on the experience of shame, levels of blaming of other people for the way they behaved was minimal, both in the questionnaire data (see Table 2) and the qualitative data. Instead scores on the TOSCA for shame self‐talk were high, and participants described the feeling of personal failure for being different.

Although the themes are distinct from each other, there was some overlap between all themes. The “phenomenology of shame” theme could be seen as providing a surface description or foreground of this emotion, beyond which the subsequent themes emerge. In contrast, “shame as a social emotion” intersected through all other themes, capturing the central essence of shame as an emotion that is about how one appears relative to others. Of the remaining themes, “failure” was probably the one that most interlinked with others. For example, when participants felt they were trapped in shame, this could lead to feelings of failure. It could be that participants felt they had to keep parts of themselves hidden due to feelings of failure and not wanting this exposed.

## DISCUSSION

### Overview

The overall aim of the current study was to investigate the psychological processes that are associated with the emergence, processing and maintenance of shame in those who engage in NSSI. Shame was present for all participants to varying degrees. The findings support existing conceptualisations of shame; that it is an inherently social emotion, often triggered by perceived failure in relation to personal or social standards and is associated with a negative self‐focus and feelings of inadequacy (Chou et al., 2018; Sheehy et al., 2019). To this end, results reflect analytical generalisability: they can be understood within existing theoretical frameworks and understandings of shame (Chenail, 2010; Smith, 2018). Furthermore, these results are congruent with previous research indicating that shame is associated with feelings of worthlessness and overexposure, and wanting to withdraw, hide or escape (Tangney et al., 2014; Van Vliet, 2008). The results build on this understanding of shame by highlighting how this emotion intersects with the difficulties faced by those who self‐injure. The social response to self‐injury was cited as a key source of shame (more so than the act of NSSI itself), creating a barrier to acceptance and enforcing perceptions of failure that often sat at the heart of shame feelings. Many participants felt trapped in their shame, through others’ responses to their difficulties, their anticipation of how others would view them and rumination. The inherent challenges of expressing and sharing these feelings with others, or controlling these feelings, contributed to the difficulty of breaking free from shame. Participants could feel both isolated with their shame, rejected by others, but also exposed and judged.

While NSSI was sometimes cited as a direct response to shame, this was not a common theme across all participants. Thus, these results only partially support suggestions that shame is a key driver of NSSI (Sheehy et al., 2019). Instead, shame represented an invasive and aversive background to experiences of NSSI. In this way, it can be seen as an important part of the distress that accompanies NSSI, and thus remains an important treatment target for people who experience NSSI.

### Limitations and future research

While participants were recruited on the basis of their shared experiences of NSSI, the broader term of “self‐harm” was typically used within the interviews. This allowed a broader range of behaviours to be considered. While behaviours referred to by participants would typically meet criteria for NSSI, it is notable that other behaviours, such as disordered eating, were also described as forms of self‐harm by some participants. The use of the term “self‐harm” therefore introduces ambiguity, and it should be noted that not all forms of self‐harm referred to by participants in the interviews would necessarily be classified as NSSI.

Although all participants reported some shame across the course of their ESM entries, this was not always their main focus (i.e. sometimes different or co‐occurring negative emotions were focused on, such as feeling low or anxious, or an urge to self‐injure), which meant there were not always large amounts of shame‐related ESM data to analyse and incorporate into the interviews. Furthermore, spontaneous responses were not available as an option due to the electronic platform used, which could have provided additional experiences of shame being captured. The questionnaire measures supported the presence of elevated shame among participants, but were relatively limited, perhaps unsurprisingly given their nomothetic nature, in capturing more nuanced characteristics of shame identified through the interview, and as such added little to the synthesis of the data.

The QUEST approach appeared beneficial. The diaries provided a focal point for semi‐structured discussion during interviews, and also acted as memory aids and prompts for participants at times, especially around the more cognitive‐focused questions. The study suggests that QUEST may be an effective method for qualitatively exploring experiences occurring within the day‐to‐day context of people's lives. Compliance rates for ESM studies in this field vary substantially from study to study (e.g., 52% in Andrewes et al., 2017; 38% in Armey et al., 2011, and 79% in Kiekens et al., 2020). The average compliance rate for diary entries in this study (approximately 60%) therefore do not appear especially low, or especially high. This suggests that the acceptability of the diary component was similar in terms of acceptability to other ESM approaches.

While this suggests the feasibility of the method, additional, focused evaluation of the approach would be beneficial.

### Clinical implications

This study illustrates how shame can be difficult for individuals to think about, focus on and talk about. Given the relational nature of the emotion, shame may be present during both individual and group therapy, and clinicians should give thought to how they discuss topics that may elicit shame, and explicitly shame itself if appropriate (Dearing & Tangney, 2011). Shame is potentially an important emotion to identify as it seems to have a powerful impact on clients’ mental health. Psychotherapies that could be useful in combating shame include Compassion Focused Therapy (CFT), which directly targets shame (Gilbert, 2009; Judge et al., 2012; Leaviss & Uttley, 2015) and has been used to treat eating disorders (Goss & Allan, 2014; Steindl et al., 2017). Relational therapies, for example, Cognitive Analytic Therapy (CAT), could be considered given the relational nature of shame, and the need for exits from shame (Jameson, 2014; Ryle & Kerr, 2020). It has been suggested that CFT and relational approaches could also be beneficial for those who self‐injure (Taylor et al., 2021; Van Vliet & Kalnins, 2011), however, research evaluating such approaches for NSSI is limited. Furthermore, systemic interventions around NSSI and eating disorders could be beneficial, as actual or anticipated responses from others (including mental health professionals) who were lacking in compassion and understanding could further exacerbate or maintain shame. Participants have often reported feeling alone with their shame and unable to articulate this to others.

## CONCLUSION

This study investigated the day‐to‐day experience of shame among people who engaged in NSSI. The result highlight how shame and NSSI are closely intertwined. While there was some indication that NSSI could be a response to shame, this was not always the case, and shame may be better understood as an ongoing emotional backdrop for these individuals, at times being a consequence of NSSI but also resulting from the ways participants were perceived and responded to by others. The results indicate that within therapeutic work with people who struggle with NSSI, attention needs to be given to the presence of shame and how this impacts on the individual.

## CONFLICTS OF INTEREST

All authors declare no conflict of interest.

## AUTHOR CONTRIBUTION

**Alexandra Brown:** Conceptualization; Data curation; Formal analysis; Investigation; Methodology; Project administration; Writing – original draft; Writing – review & editing. **Cameron Latham:** Conceptualization; Methodology; Writing – review & editing. **Adam Danquah:** Conceptualization; Supervision; Writing – review & editing. **Brendan Dunlop:** Project administration; Validation; Writing – review & editing. **Peter Taylor:** Conceptualization; Investigation; Methodology; Project administration; Supervision; Writing – review & editing.

## Supporting information

 Supplementary Material

 Supplementary Material

## Data Availability

Given the rich nature of the data it is not available to other researchers to protect participant’s confidentiality.
