# Supplementary material for: “Cover up your arms, you’re triggering people”: A Mixed‐Methods Investigation of Shame in those who Self‐Injure
Source: Psychol Psychother. 2022 Apr 11;95(3):701–16. doi: 10.1111/papt.12394 (PMC9543642; doi:10.1111/papt.12394)
Supplement: Supplementary file 1 — Supplementary Material [file PAPT-95-701-s002.docx]

# Supplementary File I

# Ecological Sampling Methodology Questions

| Timestamp: | | |
| --- | --- | --- |
| Question number | Question (answer format) | Answer |
| 1 | Since the last text, have you felt bad about yourself or something you did? (Yes/No) |  |
| 2 | How would you describe this feeling? Could you put a label on it? (Free text box): |  |
| 3 | Rate the strength of this feeling 0= not strong at all, 7= extremely strong (Likert Scale) |  |
| 4 | Did you notice this feeling anywhere in your body, for example in your chest or abdomen? (Yes/No) |  |
| 5 | If yes, please describe where you felt this (where about in your body) and what it felt like (Free text box) |  |
| 6 | What happened immediately before you felt this way? Where were you? Who were you with? Did any unwanted images or thoughts come to mind prior to this feeling? (Free text box) |  |
| 7 | What thoughts went through your mind when you first noticed this feeling? (Free text box) |  |
| 8 | Did you do anything to cope with, or try and get rid of this feeling? (Free text box) |  |
| 9 | Please feel free to write anything you would like to add in the space below. (Free text box) |  |
