# Supplementary material for: “Cover up your arms, you’re triggering people”: A Mixed‐Methods Investigation of Shame in those who Self‐Injure
Source: Psychol Psychother. 2022 Apr 11;95(3):701–16. doi: 10.1111/papt.12394 (PMC9543642; doi:10.1111/papt.12394)
Supplement: Supplementary file 2 — Supplementary Material [file PAPT-95-701-s001.docx]

**Supplementary File II**

**Initial Interview Draft Schedule**

Please note this schedule was subsequently adapted and tailored for each participant based upon their diary data.

**PART A: CAPTURE SPECIFIC EVENT**

“From looking at your diary entries, I could see there are one or two times that you experienced quite strong feelings. If it’s OK with you, we’ll be focusing on those times. Later on in the interview we’ll have an opportunity to talk about other times that seem important, and I’ll check or if there is anything else important that I have missed.”

1. Thinking about [this time], you described feeling [x], you labelled it as [y] (if they did- if not, can they put a label on this feeling?). Can you tell me a bit more about this feeling?
   1. Is this a familiar feeling to you?
   2. How does it differ compared to other negative emotions? Is it similar?
   3. (If described feeling is not labelled as shame but is consistent with shame) Some people might also refer to this feeling as “shame.” What do you think about that?
2. You mentioned you feel this (refer to location in body), what does it feel like? (If no mention of feeling shame in body- ask if they think they ever experience this).
   1. How is that different to other emotions?
3. (Read out trigger or ask about it if not reported). Can you tell me a little more about what happened here to trigger this feeling?
   1. What do you think it was about this situation that left you feeling this way?
   2. Did this feeling come on quickly following a trigger? Is that how it usually is?
   3. How do you first notice this feeling usually? What do you spot or become aware of first? Examples: thoughts, bodily sensations, feelings, memories, images, sounds, beliefs.
   4. How long does this feeling usually last? Is this similar or different to other negative emotions?
4. On this day you did this (name behaviour) to cope with the feeling – what effect did this have?
   1. Does this coping strategy always help/not help?
   2. How easy was it to do this (name coping)?
   3. Was there anything else that helped you feel better at that time?
   4. Do you use any other coping strategies? What influences your choice of coping strategy?
5. (if not already mentioned self-harm in response to Q4) Did you experience any thoughts or feelings about self-harm during this instance?
   1. How soon did these occur after you noticed the feeling?
   2. Do you think that these thoughts about self-harm were related to the feeling? In what way?
6. Did you self-harm before or after feeling this way?
7. Can you tell me a little more about what happened?
8. Do you feel your self-harm was related to the feelings you have described?
9. (if applicable) how did your self-harm affect the way you were feeling afterwards?

**PART B: FURTHER DIARY ENTRIES** (pick another entry – can repeat again if there is time left)

“I’d like to look at another time in the diaries where you also mentioned you experienced some strong feelings”

1. Thinking about [this time], you described feeling [x], you labelled it as [y] (if they did). Did this experience of this feeling seem similar or different to the example we just talked about before?
2. What was similar/different about it?
   - 1. Prompt: location in body
     2. Prompt: trigger
3. Prompt: Coping (Could use diary data to support these prompts). Did you experience any thoughts or feelings about self-harm during this instance?
4. How soon did these occur after you noticed the feeling?
5. Do you think that these thoughts about self-harm were related to the feeling? In what way?
6. Did you self-harm before or after feeling this way?
7. Can you tell me a little more about what happened?
8. Do you feel your self-harm was related to the feelings you have described?
9. (if applicable) how did your self-harm affect the way you were feeling?

**PART C: ANY OTHER EXPERIENCES**

“ We’ve talked about a few of the diary entries you filled in in more detail. Where there any points or entries in the week we’ve not covered that you think we need to talk about?”

**PART D: ENDING**

Thank you very much for talking with me today. [Check in re: how they are feeling, reiterate will receive a copy of results and diary data].
